# Infection SARS-CoV-2 chez la femme enceinte; profil épidémiologique, clinique, biologique et évolutifs, à propos de 16 cas: expérience de l’Hôpital Militaire Marocain COVID-19 de Benslimane

**DOI:** 10.11604/pamj.2021.38.384.28695

**Published:** 2021-04-20

**Authors:** Abdelhamid Benlghazi, Saad Benali, Yassine Bouhtouri, Moad Belouad, Hamza Massoudi, Jaouad Kouach

**Affiliations:** 1 Service de Gynécologie-Obstétrique, Hôpital Militaire d’Instruction Mohamed V, Université Mohammed V Rabat, Rabat, Maroc

**Keywords:** SARS-CoV-2, grossesse, femme enceinte, COVID-19, pneumonie, SARS-CoV-2, pregnancy, pregnant woman, COVID-19, pneumonia

## Abstract

Les premiers cas d'infection dus à un nouveau Coronavirus, le SARS-CoV-2 ont été enregistrés en Chine en décembre 2019. Cette maladie, désormais appelée COVID-19, a été déclarée comme pandémie par l'Organisation Mondiale de la Santé (OMS) trois mois après soit en mars 2020. Le plus souvent à l´origine d´un syndrome infectieux sans gravité, associant à différents degrés des symptômes bénins (fièvre, toux, myalgies, céphalées et éventuels troubles digestifs). Le SARS-CoV-2 peut être à l´origine de pathologies pulmonaires graves et parfois de décès. Les données sur les conséquences pendant la grossesse sont limitées. À l'heure actuelle, les données concernant l'infection par SARS-CoV-2 sont rassurantes et n'indiquent pas un nombre d'infection plus élevé ni un risque surajouté de complications chez la femme enceinte par rapport à la population générale. Quelques exceptionnels cas de mortalité maternelle existent, mais surviennent le plus souvent sur des terrains qui présentent d'autres pathologies, particulièrement la pré-éclampsie. L´objectif de notre travail est de rapporter les données cliniques, biologiques et évolutives materno-fœtales, à travers une étude rétrospective au sein de l´Hôpital Militaire Marocain COVID-19 de Benslimane, s´étalant sur une période de 3 mois allant du 21 juillet au 21 octobre 2020.

## Abstract (en)

The first cases of infection caused by new SARS-CoV-2 coronavirus were reported in China in December 2019. This disease is called COVID-19 and has been declared as a pandemic by the WHO three months after its outbreak (in March 2020). In most cases it results in non-severe infectious syndrome associated with different degrees of benign symptoms (fever, cough, myalgia, headache and potential digestive disorders). SARS-CoV-2 can cause severe lung diseased and, sometimes, it results in death. Data on its consequences during pregnancy are limited. Currently, data on SARS-CoV-2 infection are reassuring and don´t indicate a higher risk of infection or a superimposed risk of complications in pregnant women compared to the general population. A few exceptional cases of maternal mortality have been reported, but they occur, most often, in patients with other diseases, in particular pre-eclampsia. This retrospective study highlights the clinical, biological and evolutionary materno-fetal data collected in the COVID-19 Military Field Hospital of Benslimane, Morocco, over a period of 3 months, from 21^st^ July to 21 October 2020.

## Introduction

La pandémie récente de COVID-19 a entrainé une crise sanitaire mondiale sans précédent. La susceptibilité des femmes enceintes aux infections a engendré beaucoup d'interrogations sur l´éventuels complications materno-fœtales. Les modifications physiologiques liées à la grossesse font que les femmes enceintes soient plus vulnérables aux complications infectieuses et aux cardio-pulmonaires. Des taux élevés de complications maternelles ont été recensés lors des précédentes épidémies de SARS-CoV et MERS-CoV. L´objectif de notre travail est de rapporter les données cliniques, biologiques et évolutives materno-fœtales, à travers une étude rétrospective au sein de l´Hôpital Militaire Marocain COVID-19 de Benslimane, s´étalant sur une période de 3 mois allant du 21 juillet au 21 octobre 2020.

## Méthodes

**Matériels:** du 21 juillet 2020 au 21 octobre 2020, 16 femmes enceintes ont été prises en charge pour une pneumonie COVID-19 à l´Hôpital Militaire Marocain COVID-19 de Benslimane.

**Méthodes:** il s´agit d´une étude rétrospective s´étalant sur une période de 3 mois et concernant les femmes enceintes testées positives au COVID-19 qui répondent aux critères suivants; critères d´inclusion: femme enceinte; une pneumonie COVID-19 testée positive par utilisation de la rétrotranscriptase-réaction en chaine par polymérase (RT-PCR) quantitative (qRT-PCR) sur des échantillons provenant des voies respiratoires. Dans un premier temps, nous avons procédé à un triage des dossiers répondant à nos critères d´inclusion pour ressortir ceux en rapport avec une pneumonie COVID-19 chez une femme enceinte.

Une fiche d´exploitation a été dûment remplie pour chacune d´entre elles. Dans un second temps, le logiciel SPSS a été utilisé pour les analyses statistiques. Le traitement des données s´est fait en pourcentage, en moyenne ou en médiane. Le recueil des données a été effectué avec respect de l´anonymat des patientes et de la confidentialité de leurs informations. Le recueil des données évolutives après la sortie de l´hôpital a été fait par des appels téléphoniques.

## Résultats

L´âge moyen des patientes est de 32±5 ans avec prédominance de la tranche d'âge > 30 ans avec 13 patientes soit 81,3%. Aucune maladie sous-jacente telle que le diabète, l'hypertension artérielle, ou les maladies cardiovasculaires chez 75% des patientes. Par contre, il y avait 3 patientes asthmatiques sous béta-2-mimétiques à la demande et une patiente diabétique type 2 sous insuline; cependant une hypertension gestationnelle a été observée à partir de 30 semaines d´aménorrhée (SA) chez une patiente, tandis qu'une autre a développé une pré-éclampsie à 31 semaines de gestation. Ces deux patientes étaient stables pendant la grossesse.

L'exposition épidémiologique au COVID-19 a été notée chez toutes les patientes avec notion de contact avec un membre de la famille testé positif dans 75% des cas et notion de contact dans un milieu professionnel dans 25% des cas. L´âge gestationnel moyen à l´admission est de 28±7 SA. Six patientes se sont présentées avec une fièvre sans frissons, mais aucune n´a eu de fièvre élevée (température du corps > 39,5°C). Une température corporelle normale chez 6 patientes et une température entre 37,5 et 38,5°C chez 4 patientes. Les autres symptômes correspondant à une infection des voies respiratoires supérieures ont également été observés: 31% des patientes avaient une toux, 62,5% ont eu des arthro-myalgies, 43% ont déclaré des céphalées, une anosmie chez 62,5% des patientes, agueusie chez 50% et asthénie chez 62,5%. En outre, 5 patientes soit 31,3% se sont présentées avec des symptômes gastro-intestinaux évidents. Seulement deux patientes n´ont développé aucun symptôme (Tableau 1). Aucune patiente n'a développé une pneumonie sévère, nécessitant une ventilation artificielle.

**Tableau 1:** données épidémiologiques et cliniques

	Nombre de patientes	Pourcentage %
**Tranche d'âge**		
Age < 25 ans	1	6,3%
25 – 30 ans	2	12,5%
>30 ans	13	81,3%
**Histoire épidémiologique**		
Contact avec un sujet positif dans la famille	12	75%
Contact au milieu professionnel	4	25%
**Antécédents (ATCD)**		
Pas d'ATCD	12	75%
Diabète	1	6,3%
HTA	0	0%
Asthme	3	18,7%
**Age gestationnel**		
<24 SA	1	6,3%
24-28 SA	9	56,3%
>28 SA	6	37,5%
**Signes fonctionnels**		
Fièvre	6	37,5%
Température <37,5	6	37,5%
Température 37,5-38,5	4	25%
Température >38,5	6	37,5%
Anosmie	10	62,5%
Agueusie	8	50%
Toux	5	31,3%
Céphalée	7	43,8%
Dyspnée	1	6,3%
Diarrhée	5	31,3%
Ecoulement nasal	2	12,5%
Congestion nasal	2	12,5%
Arthromyalgie	10	62,5%
Asthénie	10	62,5%
Patiente asymptomatique	2	12,5%
**Signes physiques**		
SO2 > 92%	13	81,3%
SO2 92%-90%	3	18,7%
SO2 <90%	0	0%
Fréquence cardiaque 60-120 Battement/min	15	93,8%
Fréquence cardiaque >120 Battement/min	1	6,2%
Fréquence respiratoire 13-20 Cycles/min	16	100%
PAM >70mmHg	16	100%

Les complications de la grossesse qui sont apparues après l'apparition de l'infection par COVID-19 comprenaient la souffrance fœtale (chez 3 des 16 patientes) et la pré-éclampsie (chez deux cas sur 16). Les données des tests de laboratoire ont montré que deux des 16 femmes enceintes souffrant de pneumonie COVID-19 ont une lymphopénie (< 1 x 10^9^cellules par L), une patiente avait une neutropénie (< 1,5 x 10^9^cellules par L) et une patiente avec une thrombopénie (< 150 000). Une augmentation des globules blancs a été notée chez 25% des patientes, anémie chez 37,5% des patientes. Six patients avaient des concentrations élevées de protéine C-réactive (>10 mg/L) soit 37,5%. Par ailleurs la fonction rénale ainsi que les concentrations d'alanine amino-transférase (ALAT), d´aspartate amino-transférase (ASAT), de lactate déshydrogénase (LDH) et le taux de prothrombine (TP) étaient normaux chez l´ensembles des patientes (Tableau 2).

**Tableau 2:** données biologiques

	Nombre de patientes	Pourcentage %
GB <9500	12	75%
GB>9500	4	25%
Lymphopénie	2	12,5%
Neutropénie (<1500)	1	6,3%
Thrombopénie (<150 000)	1	6 ,3%
Anémie (<12g/dl)	6	37,5%
CRP positive (>10mg/l)	6	37,5%
ASAT ou ALAT élevé	0	0%
LDH > 250 U/L	12	75%
CK > 200 U/L	6	37,5%
Urée et créatinine normal	16	100%
TP >90%	16	100%
PCR positive	16	100%

Une assistance en oxygène (canule nasale) a été utilisée chez 62,5% des patientes dont 3 ont bénéficié de nébulisation au B-2-mimétiques; l´ensemble des patientes ont bénéficié d´un protocole thérapeutique: zinc 200mg/jr et vitamine C 1g/jr; un traitement antibiotique empirique a été prescrit chez 62,5% des patientes à base d´amoxicilline protégée 1g 3 fois par jour chez 3 patientes et céphalosporine de troisième génération 2g par jour chez 7 patientes. Une thrombo-prophylaxie à base d´HBPM (0,4 CC 2 fois par jour) a été utilisée chez 62,5%. Aucune patiente n´a bénéficié d´une thérapie antivirale. La PCR de contrôle au dixième jour (j 10) été négative dans 68,8% des cas; le reste été négatif au 15^e^ jour (Tableau 3). Seize naissances vivantes ont été enregistrées. Pas de mort fœtale in-utéro, ni néonatale ni asphyxie néonatale. Deux accouchements prématurés, le 1^er^ à 31 semaines d´aménorrhée pour pré-éclampsie sévère avec poids de naissance à 2100g et le 2^e^ à 33 semaines d´aménorrhée pour rupture prématurée des membranes (RPM) avec poids de naissance à 2400g. Concernant le mode d´accouchement, 8 patientes ont bénéficié d´une césarienne (3 pour utérus doublement cicatriciel; 3 pour souffrance fœtale aigüe; 2 pour pré-éclampsie) alors que les 8 autres patientes ont accouché par voie basse.

**Tableau 3:** données concernant la prise en charge thérapeutique

	Nombre de patientes	Pourcentage %
Antibiothérapie	10	62,5%
C3G 2 g/jr	7	43,7%
Amox-ac.clavul 1g x 3/jr	3	18,8%
HBPM 0,4 x2/jr	10	62,5%
Zinc 200mg/jr	16	100%
Vitamine C 1g/jr	16	100%
Paracétamol 1gx3/jr	13	81,3%
Nébulisation au salbutamol	3	18,8%
Oxygénothérapie (MHC)	7	43,7%
VNI	0	0%
VI	0	0%
PCR négative à J10	11	68,8%
PCR négative à J15	5	31,2%

## Discussion

La pandémie récente de COVID-19 a entraîné une crise sanitaire mondiale sans précédent. La vitesse avec laquelle l'infection a progressé, ainsi que l'ambigüité de son impact sur la grossesse en raison de l'absence de données scientifiques a sollicité les obstétriciens à adapter leur pratique en se basant sur des conduites pragmatiques [1]. La revue de la littérature actuelle, bien que très limitée chez la femme enceinte, semble montrer que les symptômes sont les mêmes que ceux de la population générale pour la grande majorité des femmes qui donc ne ressentiraient que de légers symptômes de rhinite ou un syndrome grippal avec potentiellement de la toux, une fièvre ou une dyspnée. Mais ces femmes peuvent également présenter des symptômes plus graves tels que la pneumonie ou le syndrome de détresse respiratoire aigüe (SDRA) comme les autres populations à risque [2].

Les femmes enceintes sont particulièrement sensibles aux pathologies respiratoires et les pneumonies graves, car elles sont dans un état immunodépressif et les changements physiologiques adaptatifs pendant la grossesse peuvent les rendre intolérantes à l'hypoxie. Par exemple, la pandémie de grippe de 1918 a causé un taux de mortalité de 2 à 6% dans l'ensemble de la population, avec 37% chez les femmes enceintes [3]. Les femmes enceintes ont été signalées comme présentant un risque accru de complications de l'infection par le virus de la grippe pandémique H1N1 2009 et avaient plus de quatre fois plus de chance d'être admises à l'hôpital que dans la population générale (risque relatif 3-4) [4].

Les données chez la femme enceinte pour le SARS-CoV-2 étant très limitées, des rapprochements peuvent être faits avec ce qui est connu dans le cadre des autres pneumopathies ou des autres coronavirus tels que le SARS-CoV ou le MERS-CoV. La pneumopathie est une cause importante de morbi-mortalité chez les femmes enceintes [5, 6]. Ainsi, les patientes développant une pneumopathie quelle qu´en soit l´étiologie devaient, dans une étude assez ancienne, dans 25% des cas être hospitalisées dans des unités de soins intensifs avec une assistance ventilatoire [7]. En effet, comme pour d´autres maladies infectieuses, les changements physiologiques maternels normaux accompagnant la grossesse avec une modification de l´immunité [8, 9] et des changements cardio-pulmonaires pourraient être à l´origine de la plus grande sensibilité et de l´augmentation de la gravité clinique de la pneumopathie [10]. En 2009, les femmes enceintes représentaient 1% des patients infectés par le virus H1N1 mais elles représentaient 5% de tous les décès liés au virus. Les patientes avec des pneumopathies seraient également plus à risque de rupture prématurée des membranes, d´accouchements prématurés, de morts fœtales *in utero*, de retards de croissance intra utérins et de décès néonataux [10].

Nous rapportons les données cliniques, biologiques et évolutives de seize femmes enceintes avec une pneumonie COVID-19 confirmée au laboratoire. Au 1^er^ décembre 2020, aucune des seize patientes n'avait développé de pneumonie grave ou n'était décédé. D'après nos résultats concernant ces 16 patientes, il n'existe actuellement aucune preuve suggérant que le développement d'une pneumonie COVID-19 au cours de la grossesse pourrait entraîner l'apparition des formes graves. Dans l´étude de Chen *et al*. [1] il y avait 9 patientes enceintes au 3^e^ trimestre testées positives pour le SARS-CoV-2. Parmi elles, 7 ont présenté de la fièvre, 4 de la toux, 3 des myalgies, 2 une odynophagie et 2 des malaises. Cinq présentaient une lymphopénie, 3 des perturbations du bilan hépatique. Aucune n´a développé de pneumonie sévère et aucune n´est décédée à l´heure actuelle. Elles ont toutes bénéficié d´une césarienne. Zhu *et al*. [11] ont analysé rétrospectivement les caractéristiques cliniques de 9 mères dans 5 hôpitaux du Hubei, parmi ces femmes, 4 ont présenté des symptômes dans les 4 jours avant l´accouchement, 2 le jour de l´accouchement et 3 par la suite. Dans la majorité des cas, les symptômes maternels étaient: la fièvre et la toux. Six enfants sont nés prématurés.

Selon notre étude, la pneumonie COVID-19 de ces femmes comprenait une fièvre, anosmie, agueusie, arthro-myalgie et l´asthénie, alors que les symptômes les moins fréquents sont la toux, diarrhée, dyspnée et congestion nasale et on note la présence de deux cas asymptomatiques. L´état hémodynamique et respiratoire des patientes été stable à l´admission avec saturation en oxygène >92% chez l´ensemble des patientes. Sur le plan biologique, on observe fréquemment, une ascension des protéines de l'inflammation, une neutropénie concernant surtout les lymphocytes et une thrombopénie. Les explorations radiologiques notamment le scanner thoracique bien qu'elles ne soient pas obligatoires pour établir le diagnostic peuvent être d'apport pour orienter la prise en charge et ne doivent jamais être évités en raison de la grossesse car le pronostic vital maternel peut être mis en jeu dans les formes sévères. Ainsi et pour être pratique, une évaluation minutieuse de l'état clinique, la nécessité d'une hospitalisation voire même l'indication d'une admission dans une unité de soins intensifs (USI) peuvent être évalués à l'aide de scores cliniques dont les valeurs ont été adaptées à une population obstétricale [12].

Dans notre série, les bilans biologiques ont objectivé que la lymphopénie; la thrombopénie et la neutropénie sont également susceptibles de se produire. Avec une protéine C-réactive (CRP) positive (>10 mg/l) chez 6 patientes, aucune patiente n´a bénéficié d´un scanner thoracique vu la stabilité clinique de l´ensemble des cas. La prise en charge des patientes malades de COVID-19 ne diffère pas du reste de la population en dehors de la pathologie obstétricale. Ainsi, les patientes pauci ou asymptomatiques peuvent être suivies en ambulatoire moyennant des contrôles téléphoniques dans un but d'assurer la continuité du suivi durant cette période. Pour les patientes symptomatiques et qui présentent une défaillance notamment respiratoire, une hospitalisation dans un service COVID-19 voire même en unité de soins intensifs peut être justifiée.

La nécessité d'un traitement anticoagulant préventif est discutée, mais les données non publiées rapportent des taux élevés de complications thrombo-emboliques et ouvrent la question sur l'administration prophylactique systématique chez toute patiente enceinte hospitalisée avec une infection prouvée à SARS-CoV-2. Dans notre étude, la thrombo-prophylaxie à base d´héparine de bas poids moléculaire (0,4 cc en sous cutané 2 fois par jour) a été administrée chez les patientes avec un IMC > 30 et/ou un syndrome infectieux et/ou CRP > 10 et/ou GB > 9500. D'autres molécules (chloroquine, azithromycine, etc.) sont actuellement en cours d'essai clinique. Les bénéfices de ces traitements restent à prouver mais ces médicaments couramment utilisés au sein du Centre Hospitalier Universitaire (sous surveillance stricte notamment des effets indésirables) présentent un profil pharmacologique rassurant durant la grossesse, sans effets tératogènes connus [13]. Dans notre étude, une antibiothérapie a été administrée chez les patientes avec une un syndrome infectieux et/ou CRP >10 et/ou GB > 9500.

Du point de vue obstétrical, la voie d'accouchement ne devrait pas être influencée par la présence d'une infection à SARS-CoV-2, mais guidée par les indications obstétricales habituelles et l'état clinique de la patiente. Les efforts expulsifs peuvent être compromis par la gêne respiratoire et doivent être écourtés par l'utilisation d'un instrument (forceps, ventouse, spatule...). Le recours à une césarienne peut être indiqué chez les patientes qui présentent une détresse respiratoire (indication de sauvetage maternel). En cours de travail, une surveillance du rythme cardiaque fœtal et de l'état hémodynamique maternel doit être constante comme habituellement. Sur le plan analgésique, une anesthésie péridurale devrait être privilégiée pour diminuer le risque d'intubation liée à une anesthésie générale en cas de césarienne en urgence. La thrombopénie souvent observée en cas d'infection COVID-19 justifie un contrôle du taux des plaquettes de façon régulière notamment à l'entrée en salle de travail. Évidemment, des précautions particulières doivent être prises au sein du personnel soignant notamment minimiser le nombre de personnes en contact avec une patiente infectée et insister sur le port de matériel de protection tel que blouses et masques. Dans notre établissement, nous préconisons des mesures supplémentaires par port de masques ultra-filtrants (FFP2) et de lunettes lors de l'accouchement, en raison du risque d'aérosolisation.

Dans notre étude tous les accouchements ont été réalisés après guérison des patientes avec un test PCR négatif chez l´ensemble des patientes et les indications de césarienne étaient purement obstétricales. Les auteurs concluent que l´infection maternelle périnatale peut avoir des conséquences néfastes sur les issues obstétricales et sur les nouveau-nés entraînant notamment des détresses respiratoires, des anomalies biologiques des accouchements prématurés et même un décès. Ils émettent l´hypothèse que l´hypoxémie chez la mère puisse être responsable de l´hypoxie fœtale à la naissance et de l´accouchement prématuré [13]. La prématurité a été notée dans deux cas l´un pour prééclampsie et l´autre pour chorioamniotite ainsi que la souffrance fœtale aigüe a survenu au cours du travail chez des patientes déclarées guéries de COVID-19 donc on ne peut pas lier l´hypoxie fœtale directement au COVID-19.

**Limitations:** parmi les limites de notre étude, la plus importante est le petit nombre des patientes hospitalisé dans notre hôpital pour infection par SARS-CoV-2 entrainant ainsi l´impossibilité de faire un suivie complet par un examen clinique et par l´échographie selon un rythme précis des patientes après la guérison à la recherche d´éventuels complications fœtal et d´organiser une prise en charge adéquate.

## Conclusion

En résumé, les symptômes les plus fréquents chez les femmes enceintes souffrant de pneumonies COVID-19 étaient diverses, les principaux symptômes étant l´anosmie, l´agueusie, l´asthénie et la fièvre. D'après nos résultats concernant ces 16 patientes, il n'existe actuellement aucune preuve suggérant que le développement d'une pneumonie COVID-19 au cours de la grossesse pourrait entraîner l'apparition des formes graves.

### Etat des connaissances sur le sujet

- Il s'agit d'une pandémie mondiale avec une mortalité élevée dans le monde entier;
- Les études publiées sur le l´infection par SARS-CoV-2 au cours de la grossesse sont peu nombreuse;
- Il existe peu de données sur le retentissement périnatal après une infection par COVID-19 pendant la grossesse ou le postpartum.

### Contribution de notre étude à la connaissance

- Elle décrit les caractéristiques épidémiologiques, cliniques, biologiques et évolutifs des patientes enceintes admises;
- Notre étude donne un aperçu sur l´impact de l´infection SARS-CoV-2 sur la femme enceinte.
